# Remotely Monitored Gamification and Social Incentives to Improve Glycemic Control Among Adults With Uncontrolled Type 2 Diabetes (iDiabetes): Protocol for a Randomized Controlled Trial

**DOI:** 10.2196/14180

**Published:** 2019-11-20

**Authors:** Michael Fortunato, Joseph Harrison, Ai Leen Oon, Dylan Small, Victoria Hilbert, Charles Rareshide, Mitesh Patel

**Affiliations:** 1 University of Pennsylvania Philadelphia, PA United States; 2 Penn Medicine Nudge Unit Philadelphia, PA United States; 3 Crescenz Veteran Affairs Medical Center Philadelphia, PA United States

**Keywords:** behavioral economics, gamification, social incentives, diabetes, glycemic control weight, physical activity, remote monitoring, wearable devices

## Abstract

**Background:**

Type 2 diabetes is a significant cause of morbidity and mortality in the United States. Lifestyle modifications including increasing physical activity and losing weight have been demonstrated to improve glycemic control. However, most patients struggle to make these changes. Many stakeholders are interested in using gamification and social incentives to increase engagement in healthy behaviors. However, these approaches often do not appropriately leverage insights from behavioral economics that could be used to address predictable barriers to behavior change.

**Objective:**

This study aimed to describe the protocol for the *Influencing DIabetics to Adapt Behaviors related to Exercise and weighT by Enhancing Social incentives* (iDiabetes) trial, which aimed to evaluate the effectiveness of gamification interventions that leverage insights from behavioral economics to enhance supportive, competitive, or collaborative social incentives to improve glycemic control, promote weight loss, and increase physical activity among overweight and obese adults with type 2 diabetes.

**Methods:**

We are conducting a one-year four-arm randomized controlled trial of 361 overweight and obese patients with type 2 diabetes and a glycated hemoglobin (HbA_1c_) level ≥8.0. Wireless weight scales and wearable devices are provided to remotely monitor weight and physical activity and transmit data to the study team. Patients are recruited by email, following which they establish a baseline measure of weight, daily step count, HbA_1c_ level, and low-density lipoprotein cholesterol level and then repeat these measures at 6 and 12 months. The control arm receives no other interventions. Patients randomized to one of the three intervention arms are entered into a game designed using insights from behavioral economics to enhance supportive, competitive, or collaborative social incentives. To examine predictors of strong or poor performance, participants completed validated questionnaires on a range of areas including their personality, risk preferences, and social network.

**Results:**

Enrollment of 361 patients was completed in January 2019. Results are expected in 2020.

**Conclusions:**

The iDiabetes trial represents a scalable model to remotely monitor the daily health behaviors of adults with type 2 diabetes. Results from this trial will help provide insights into how to improve management of patients with type 2 diabetes.

**Trial Registration:**

ClinicalTrials.gov NCT02961192; https://clinicaltrials.gov/ct2/show/NCT02961192

**International Registered Report Identifier (IRRID):**

DERR1-10.2196/14180

## Introduction

### Background

Type 2 diabetes is a significant cause of morbidity and mortality in the United States [1,2]. Lifestyle modifications including increasing physical activity and losing weight have been demonstrated to improve glycemic control [3,4]. However, changing daily health behaviors can be challenging for many patients, especially those who start with lower levels of motivation. Recently, many stakeholders have become interested in using mobile technologies to passively monitor these daily behaviors and to deploy interventions at a broader scale [5,6].

Gamification is the use of game design elements in nongame settings and is increasingly being used in interventions to promote healthy behaviors [7]. Although gamification is used widely, it is often not designed to incorporate theories from health behavior [8-11]. Behavioral economics is a field that incorporates principles from psychology and economics to help us understand why individuals make decisions that are predictably irrational [12,13]. For example, individuals tend to be more motivated to avoid losses than gain an equivalent reward [14-16], by immediate rather than delayed gratification [17], and they tend to avoid the feeling of regret [18]. In a randomized trial, members of our group found that insights from behavioral economics could be embedded within gamification design to enhance social incentives such as accountability, peer support, and collaboration to significantly increase physical activity among families in the community [19]. We have also conducted a pilot study to demonstrate how these approaches could be used to promote weight loss [20].

### Objective

The objective of this article was to describe the protocol for the *Influencing DIabetics to Adapt Behaviors related to Exercise and weighT by Enhancing Social incentives* (iDiabetes) trial, which aimed to evaluate the effectiveness of gamification interventions that leverage insights from behavioral economics to enhance either supportive, competitive, or collaborative social incentives to improve glycemic control, promote weight loss, and increase physical activity among overweight and obese adults with type 2 diabetes. This builds upon on our previous work by testing more types of social incentives and implementing them with a more high-risk population. The trial recruited patients from Penn Medicine, an academic health system in Philadelphia, and used a Web-based platform at the University of Pennsylvania, called Way to Health [21], which facilitated virtual recruitment, Web-based informed consent, automated study communication, and remote monitoring of behavior.

## Methods

### Study Design

iDiabetes is a 4-arm randomized controlled trial with a 1-year intervention period. The trial was conducted using Way to Health [21], an automated information technology platform at the University of Pennsylvania that integrates wireless devices, conducts clinical trial randomization and enrollment processes, delivers messaging (via text or email), delivers self-administered surveys, automates payment transfers, and securely captures data for research purposes. This platform has been used to run over 100 clinical trials including several by our group focusing on physical activity and weight loss [8,15,16,19,20,22-25]. All participants received US $25 for completing laboratory testing to assess baseline hemoglobin A1c (HbA_1c_) and low-density lipoprotein cholesterol (LDL-C) during the enrollment process. Participants who were eligible and completed enrollment into the study received an additional US $25. Participants received US $50 to obtain laboratory tests (HbA_1c_ and LDL-C) and conduct a virtual weigh-in at home via FaceTime (Apple Inc.) or Skype (Microsoft Inc) after 6 months and 12 months, similar to prior work [20]. Participants were randomly assigned to the control arm or 1 of 3 gamification intervention arms designed to enhance supportive, competitive, or collaborative social incentives. Data on participant characteristics were collected through validated questionnaires. The University of Pennsylvania Institutional Review Board approved the study.

### Participant Recruitment

Potential participants were identified from EPIC, the electronic health record at Penn Medicine, by using Penn Data Store (the health system’s clinical data warehouse) and Clarity, an EPIC reporting database. The study team sent email invitations, letters, and made phone calls introducing the study and its eligibility criteria with instructions on how to learn more and begin the enrollment process online. Recruitment occurred from January 23, 2017, to January 7, 2019.

Participants were eligible for the program if they were aged between 18 and 70 years, they were able to read and provide informed consent to participate, they had a diagnosis of type 2 diabetes with an HbA_1c_ level of 8.0 or greater within the last 90 days, they had a self-reported body mass index of 25 or greater, and they owned a smartphone or tablet compatible with the wearable device and wireless weight scale. Participants were excluded if there was a condition that made their participation unfeasible (eg, inability to provide informed consent and illiteracy or inability to speak, read, and write English), if there was a condition that made participation unsafe (eg, pregnancy, previous diagnosis of an eating disorder, or history of unsafe weight loss practices), or if he or she was already enrolled in another study targeting physical activity, weight loss, or glycemic control or any other medical conditions or reasons because of which the participant would be unable to complete the 1-year program.

### Participant Enrollment

Interested participants were instructed to visit the study website to create an account, review and complete informed consent, and complete the eligibility survey. Eligible participants who had not recently had blood tests were then instructed to obtain baseline HbA_1c_ and LDL-C laboratory tests that were conducted at either a qualifying Penn Medicine facility or a Quest Diagnostics laboratory. Participants were also instructed to complete a series of assessments including surveys and validated questionnaires described in Table 1. These included a baseline survey assessing sociodemographic and health characteristics, the domain-specific risk-tasking survey collecting data related to risk preferences [26], personality type (Big Five Inventory) [27], and the Medical Outcomes Study Social Support survey [28]. The qualitative assessment at the end of the study is conducted through an online survey and participants are asked to rate their satisfaction with the devices, interventions, and study overall by using Likert scales (strongly agree, agree, neutral, disagree, and strongly disagree). Participants are also asked to respond to open-ended prompts to describe ways in which their experience helped or did not help them to lose weight, increase physical activity, and manage their diabetes.

**Figure 1:**
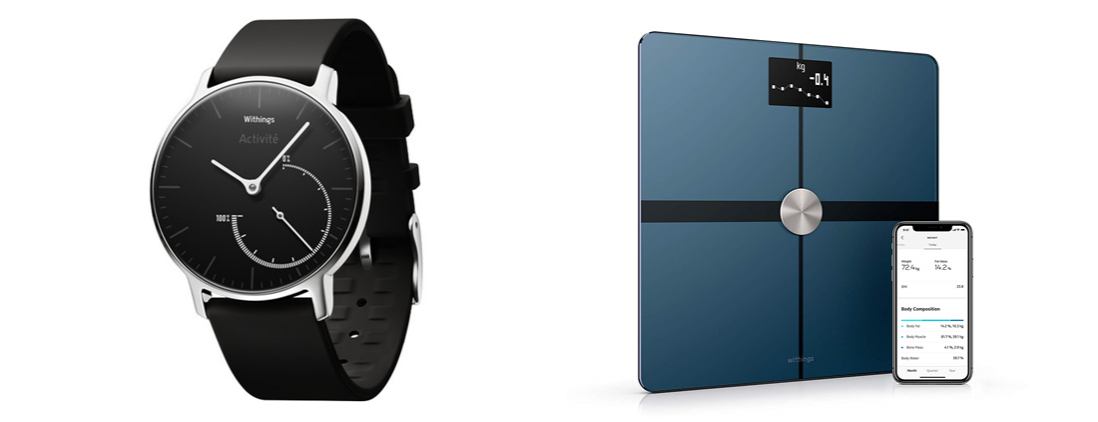
Depiction of the wireless devices used by participants.

After baseline assessments were completed, participants were mailed a wearable device (Withings Steel) and were given instructions on how to authorize the device to send data to the Way to Health technology platform (Figure 1). This wearable device tracked physical activity (daily step count) and sleep (minutes of total sleep, light sleep, and deep sleep). These types of devices have been demonstrated by our previous study to be accurate for tracking step counts [29]. The wearable device was waterproof and had a battery that lasted about 6 months. Participants were provided with a replacement battery at the beginning of the study and, if necessary, were mailed a new battery later. The study team was available on phone to help participants set up the wearable device.

**Table 1:** Participant survey assessments.

Domain	Questionnaire	Questions (n)	Baseline	6 months	12 months
Baseline	—^a^	—	✓	—	—
Social support	Medical outcomes study social support	19	✓	—	—
Risk preferences	Domain-specific risk-tasking survey	30	✓	—	—
Personality	Big Five	44	✓	—	—
Qualitative survey	—	—	—	—	✓

^a^Not applicable.

### Baseline Step Count

Once the participants’ wearable device was setup and connected to the study, they were asked to get used to wearing the device for several weeks. During this period, a baseline step count was estimated using the second week of data—a method used in previous work [16,19]. The first week of data was ignored to diminish the potential upward bias of the estimate from higher activity during initial device use. To prevent potential mismeasurement, we ignored any daily values less than 1000 steps because evidence indicates that these values are unlikely to represent capture of the actual activity [30,31]. If less than 4 days of data were available during the second week, the patient was contacted to inquire about any device issues, and the run-in period was expanded until at least four days of data were captured.

### In-Person Visit and Randomization

After a baseline step count had been established, the participant was scheduled for an in-person visit with the study team to complete the enrollment process. During this visit, all participants received education on the importance of diet and physical activity for weight loss and glycemic control. The participants were provided with a wireless weight scale and had a baseline weight measured (Figure 1). As weight can fluctuate based on time of day and clothing, we monitored for weights taken at home that were >5 lbs different. In those cases, the study team reached out to participants to clarify the most accurate weight to use as his or her baseline. At the in-person visit, participants were then randomized to one of the study arms. A participant was considered ready to be randomized once he or she had completed all surveys, established a baseline step count, completed baseline laboratory work for HbA_1c_ and LDL-C, and had come in for an in-person study visit to establish a baseline weight measurement. Randomization was conducted electronically using block sizes of 4 groups with 3 participants per group. The first participant in the group was randomly assigned to an arm, and the next 2 participants were assigned to fill that group. In most cases, the participants in a group did not previously know each other. However, if 2 participants did know each other and wanted to join together, then they were randomized together as either groups of 2 or 3 persons. Participants were informed of their arm assignment during the in-person visit with the opportunity to ask any questions related to the intervention design.

All investigators, statisticians, and data analysts were blinded to arm assignments until the study and analysis were completed.

### Interventions

Participants in the control arm used the devices but received no other interventions and were not asked to conduct goal setting. Participants randomized to 1 of the 3 gamification arms conducted goal setting during the in-person visit. This included selecting a HbA_1c_ reduction goal (1.5%, 2%, or 2.5%), a weight loss goal (6%, 7%, or 8% of their baseline weight), or an increase in daily step counts (33%, 40%, or 50% greater than baseline, or any step goal that is at least 1500 steps above their baseline). A number of design considerations were incorporated to help participants achieve their goals while minimizing risks. First, the weight loss goal was set for a gradual decline over the first 26 weeks and then to maintain that level during the next 26 weeks. Participants were given a weight target each week, and if not achieved, the target remained the same for the following week. Second, participants had a 4-week ramp up toward their daily step goal. For example, a participant with a baseline of 6000 steps per day and goal of 8000 steps per day was asked to achieve goals of 6500, 7000, 7500, and 8000 for each of the first 4 weeks of the study. Then, they were asked to maintain the 8000 steps per day goal for the remainder of the study. Finally, the HbA_1c_ goal was set for the 6-month time point and was expected to be maintained through 1 year.

**Figure 2:**
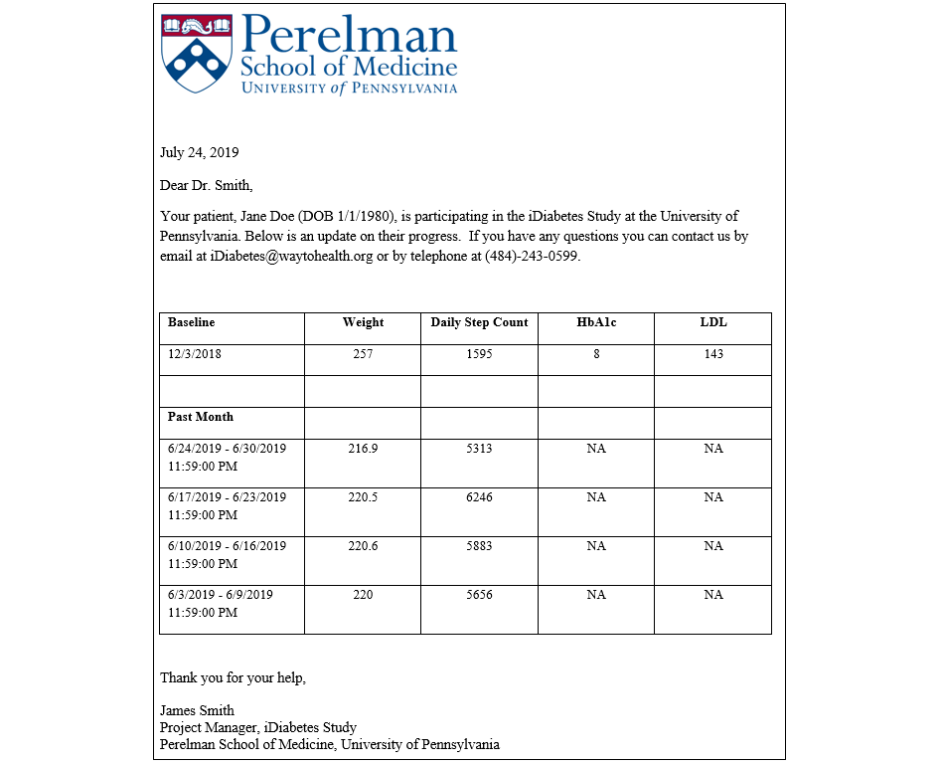
Example of a physician letter.

**Figure 3:**
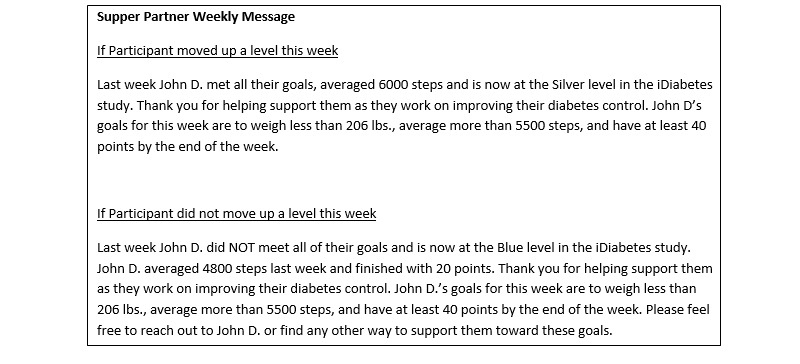
Example of support partner messages.

Participants in the gamification arms were entered into an intervention approach based on prior work that used points and levels designed to incorporate insights from behavioral economics [19,20]. First, participants were asked to sign a precommitment pledge to strive to achieve their goals during the study. Precommitment has been demonstrated to motivate behavior change [32,33]. Second, at the beginning of each week, the participant received 70 points (10 for each day that week). If the participant did not weigh-in, they lost 10 points from their balance. This leverages loss aversion, which has demonstrated that loss framing is more effective at motivating behavior change than gain framing [14-16]. Third, at the end of each week, if participants had at least 40 points, achieved their weekly weight target, and averaged at or above their daily step goal, they were moved up a level (levels from lowest to highest: blue, bronze, silver, gold, and platinum). If not, participants were dropped a level. This design creates achievable goal gradients—the notion that the next highest level was attainable—and a sense of social status and progression through the game. All participants begin at the silver level, so that they will feel the loss of dropping to bronze if they do not achieve enough points in the first week. Participants who finished in the platinum level received a trophy, and participants who finished in the gold level received a medal. Fourth, participants got a new set of 70 points every Monday. This design leverages the *fresh start effect* that is the tendency for aspirational behavior around temporal landmarks such as the beginning of the year, month, or week [34]. Fifth, to help re-engage participants who are struggling to meet their goals at months 3, 6, and 9 (defined as being in the blue or bronze levels of the game), the study coordinators called them to inquire about their progress in the study, reset them to the silver level, and offered them the opportunity to readjust their goals based on their initial options. Sixth, the participants’ primary care physician was mailed a monthly report with data on their change in weight, step goals, HbA_1c_, and LDL-C (Figure 2). A copy of this letter was also mailed to the participant. Finally, the game varied based on the social incentive arm described as follows:

1. In the supportive social incentive arm, participants were asked to identify a family member or friend to be their support sponsor. This sponsor is encouraged to support the participant in their progress during the study. A weekly report is sent by email to the sponsors with the participants’ performance including their points and level (Figure 3).
2. In the competitive social incentive arm, participants are placed into a group of 3 participants. These participants typically did not know each other before the study but were introduced to each other by email at the beginning of the intervention. At the end of each week, the participants receive an email with a leaderboard that ranks them on their cumulative points in the study thus far and also displays their level. In the event of a tie in total cumulative points, the participants will be secondarily ranked on level. This feedback may help to induce participants to compete for the top spot among the group.
3. In the collaborative social incentive arm, participants are placed into a group of 3 participants as a team. These participants typically did not know each other before the study but were introduced to each other by email at the beginning of the intervention. Each day, one of the members of the group is randomly selected to represent his or her team for that day, and that information is shared with the entire group. If the participant selected weighed in on the previous day, the team keeps their points. If he or she did not, then the team is told that they lost 10 points. In this design, each person is accountable to the others in the team, and this may induce a collaborative effort to meet their daily goals. The entire team moves up a level only if the team has at least 40 points by the end of the week.

### Outcome Measures

The primary outcomes were change in HbA_1c_, change in weight in pounds, and change in mean daily steps from baseline to the end of the 1-year study. Secondary outcomes include the change in LDL-C levels from baseline to end of the 1-year study, and change in mean daily steps, weight in pounds, and HbA_1c_ from baseline to the 6-month time point of the study.

### Power

We estimated that a sample size of 360 participants (90 per arm) will provide at least 80% power using a conservative Bonferroni adjustment of the type I error rate with a 2-sided alpha of .017 and accounting for a dropout rate of 10% to detect (1) an 1100-step change in physical activity with a standard deviation of 2000 steps; (2) a 6-lb change in weight with a standard deviation of 10 lbs; and (3) a 0.8% change in HbA_1c_ with a standard deviation of 1.5%. These values are based on prior work [19,20,35,36]. This trial has been powered for 2 phases of hypothesis testing. In the first phase, we will compare each of the 3 intervention arms with control. In the second phase, we will compare successful intervention arms with each other. We expect that the magnitude of difference between intervention arms will be less than that of successful intervention arms compared with control. For this second phase of analyses, we will use a conservative Bonferroni adjustment of the type I error rate with a 2-sided alpha of .017 to adjust for up to 3 comparisons. As only intervention arms that demonstrated a significant difference with the control are compared with each other in the second phase, the overall family-wise error rate of this 2-phase procedure is controlled at 0.05 [37].

### Statistical Analysis

All analyses will be performed using intention-to-treat. For the main analysis, we will use multiple imputation for missing data. Similar to prior work [16,19], for missing step data, including values less than 1000 steps per day, we will perform 5 sets of imputations, and results will be combined using Rubin’s standard rules [38]. We will perform sensitivity analyses using available data without imputation. The primary analysis will fit a mixed effect regression model to evaluate changes in outcome measures adjusted for each participant baseline measure, time at the observation level using calendar month fixed effects, and participant random effects. Secondary analyses will fit mixed effects regression models adjusted for other variables of interest such as participant characteristics.

To understand predictors of response to the interventions, exploratory analyses will fit mixed effects regression models to evaluate associations of participant characteristics or behaviors with strong or poor performance in the outcome measures. In addition, we will use latent class analysis of the baseline variables to identify classes of participants and compare differences in their performance across the arms. We will also conduct an exploratory qualitative evaluation of the survey’s free text responses by using grounded theory to identify themes reported by the participants.

## Results

**Figure 4:**
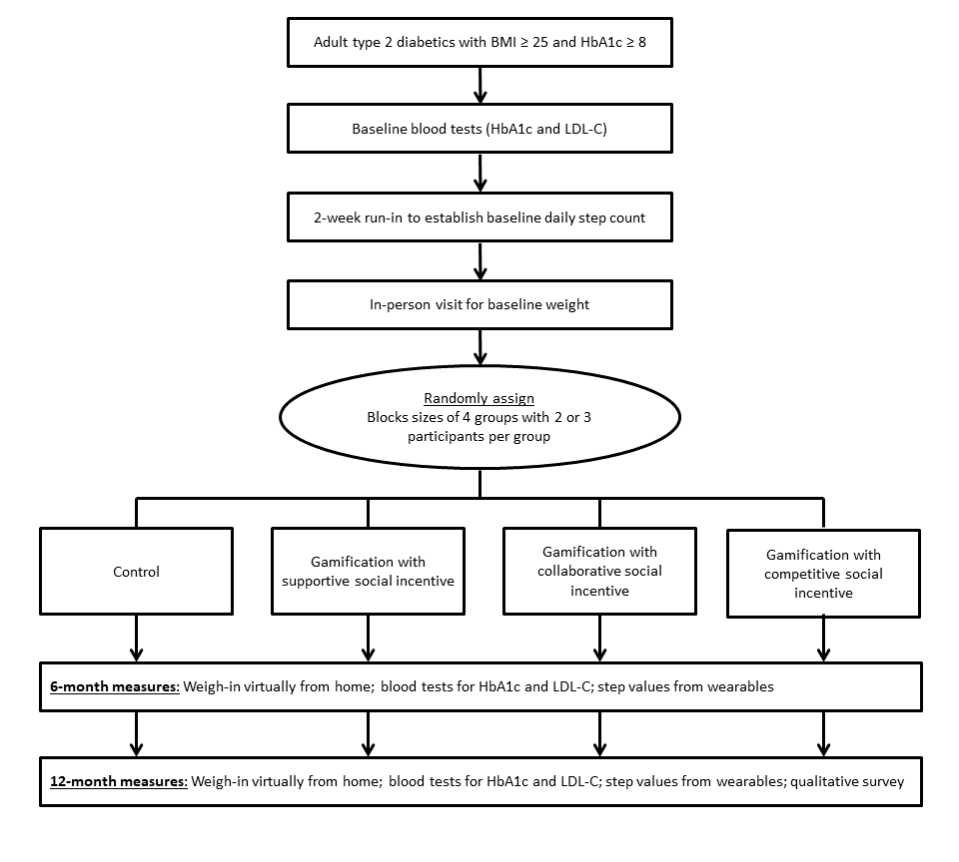
Study flow diagram of participants randomized into the *Influencing DIabetics to Adapt Behaviors related to Exercise and weighT by Enhancing Social incentives* trial. BMI: body mass index; HbA_1c_: glycated hemoglobin; LDL-C: low-density lipoprotein cholesterol.

The study flow diagram is depicted in Figure 4. Over a 2-year period, 361 participants were randomized into the trial. Among the approximately 10,000 individuals identified in the electronic health record and invited to participate, 1420 created an account on Way to Health and were assessed for eligibility. Reasons for exclusion included ineligibility (168), declining informed consent (61), and not completing all enrollment steps before recruitment closed (651). The trial will conclude in January 2020, and analyses will be reported separately.

## Discussion

### Overview

Daily behaviors related to management of type 2 diabetes have typically been challenging to address because they occur within the everyday lives of patients and not during in-person visits with a clinician. The iDiabetes trial uses remotely monitored devices to test a scalable approach to monitor these behaviors and deploy interventions. Insights from behavioral economics are incorporated within the gamification interventions to address predictable barriers to behavior change. Social incentives, which are the influencers that motivate individuals to adjust their behaviors based on social ties and connections [39-41], are compared across different designs including supportive, competitive, and collaborative types. We will also explore whether data from validated assessments completed by participants can identify predictors of response to interventions.

### Strengths and Limitations

This study has several limitations. First, only a small portion of the individuals invited enrolled into the trial, and this may limit generalizability. Second, the control arm did not receive daily messaging; therefore, we cannot disentangle the impact of the gamification interventions with daily messaging. Third, we are evaluating physical activity using step counts and do not have other measures of physical activity or exercise. Finally, we are unable to measure variations in the amount of support, competition, or collaboration for each participant in those respective arms.

This study also has several strengths. First, although gamification is used widely by insurance and workplace wellness programs, these designs often do not incorporate principles from theories of health behavior. Lessons from this study could help to inform the design of those programs to increase effectiveness. Second, insights from interventions among diabetics could be applied to patients with other chronic conditions that may benefit from changes in physical activity or weight. Third, this trial was conducted through a remote-monitoring approach that could be scaled more broadly at a lower cost than a more personnel-intensive approach. Finally, our exploratory analysis will enable us to design more targeted interventions in the future by understanding which participants respond best to each of the interventions.

### Conclusions

The iDiabetes study is one of the first evaluations of behaviorally designed gamification in a high-risk patient population. This trial has demonstrated that it is feasible to conduct a remotely monitored intervention, and the findings could help us to understand how to improve the management of adults with type 2 diabetes.

## Abbreviations

HbA_1c_: glycated hemoglobin
iDiabetes: Influencing DIabetics to Adapt Behaviors related to Exercise and weighT by Enhancing Social incentives
LDL-C: low-density lipoprotein cholesterol
